# Differential Effect of *Helicobacter pylori* Eradication on Time-Trends in Brady/Hypokinesia and Rigidity in Idiopathic Parkinsonism

**DOI:** 10.1111/j.1523-5378.2010.00768.x

**Published:** 2010-08

**Authors:** Sylvia M Dobbs, R John Dobbs, Clive Weller, André Charlett, Ingvar T Bjarnason, Andrew J Lawson, Darren Letley, Lucy Harbin, Ashley B Price, Mohammad A A Ibrahim, Norman L Oxlade, James Bowthorpe, Daniel Leckstroem, Cori Smee, J Malcolm Plant, Dale W Peterson

**Affiliations:** * Psychological Medicine and Pharmaceutical Sciences, King’s College LondonLondon, UK; † Department of Gastroenterology, Guy’s, King’s, St Thomas’ School of MedicineLondon, UK; ‡ Statistics Unit, Health Protection AgencyLondon, UK; § Laboratory of Gastrointestinal Pathogens, Health Protection AgencyLondon, UK; ¶ Nottingham Digestive Diseases Centre Biomedical Research Unit, University HospitalNottingham, UK; ** Department of Histopathology, Northwick Park and St. Mark’s Hospitals, Imperial CollegeLondon, UK; †† Department of Immunology, Guy’s, King’s, St Thomas’ School of MedicineLondon, UK; ‡‡ School of Life Sciences, University of HertfordshireHatfield, Hertfordshire, UK

**Keywords:** *H. pylori* eradication, idiopathic parkinsonism, treatment failure, anti-nuclear antibody, low-density infection, small intestinal bacterial overgrowth

## Abstract

**Background:**

We examine the effect of eradicating *Helicobacter* in idiopathic parkinsonism (IP). Marked deterioration, where eradication-therapy failed, prompted an interim report in the first 20 probands to reach de-blinding. The null-hypothesis, “eradication has no effect on principal outcome, mean stride length at free-walking speed,” was rejected. We report on study completion in all 30 who had commenced post-treatment assessments.

**Methods:**

This is a randomized, placebo-controlled, parallel-group efficacy study of eradicating biopsy-proven (culture and/or organism on histopathology) *Helicobacter pylori* infection on the time course of facets of IP, in probands taking no, or stable long-*t*½, anti-parkinsonian medication. Persistent infection at de-blinding (scheduled 1-year post-treatment) led to open active eradication-treatment.

**Results:**

Stride length improved (73 (95% CI 14–131) mm/year, *p* = .01) in favor of “successful” blinded active over placebo, irrespective of anti-parkinsonian medication, and despite worsening upper limb flexor rigidity (237 (57–416) Nm × 10^−3^/year, *p* = .01). This differential effect was echoed following open active, post-placebo. Gait did not deteriorate in year 2 and 3 post-eradication. Anti-nuclear antibody was present in all four proven (two by molecular microbiology only) eradication failures. In the remainder, it marked poorer response during the year after eradication therapy, possibly indicating residual “low-density” infection.

We illustrate the importance of eradicating low-density infection, detected only by molecular microbiology, in a proband not receiving anti-parkinsonian medication. Stride length improved (424 (379–468) mm for 15 months post-eradication, *p* = .001), correction of deficit continuing to 3.4 years. Flexor rigidity increased before hydrogen-breath-test positivity for small intestinal bacterial overgrowth (208 (28–388) Nm × 10^−3^, *p* = .02), increased further during (171 (67–274), *p* = .001) (15–31 months), and decreased (136 (6–267), *p* = .04) after restoration of negativity (32–41 months).

**Conclusion:**

*Helicobacter* is an arbiter of progression, independent of infection-load.

Determining what is driving idiopathic parkinsonism (IP) necessitates stepping-back to consider the whole clinical entity. The prevailing paradigm is that a hit-and-run insult leads to cold degeneration of dopaminergic neurons. The broad epidemiological brush reveals an interesting cluster of associations: familial aggregation, rural living, farm exposure, source of drinking water (wells and rivers), and the apparent protective effect of tea drinking [1]. We employed the classical spousal approach to environmental causality. Marked, multifarious, relevant differences (physiological/psychomotor/dermatological), between spouses of IP probands and control couples were difficult to explain by selective mating or learned/reactive behavior (for review, see [2]). That is the spouses appear a short, but highly significant, distance down the pathway. This and relative lymphopenia [3], in a large group of IP probands and spouses, suggested adult transmission of a primer. Moreover, half of the probands and a third of their spouses had chronic bowel abnormality [2]. There is both systemic and nigro-striatal immune activation in IP. Indeed, we found biological gradients between measures of IP and circulating markers of inflammation. The concept of a systemic infection primer emerged.

Back in 1965, Strang [4] observed an excess of previously diagnosed peptic ulcer in IP. However, the link had remained unexplored after recognition of *Helicobacter pylori*-associated gastritis in 1983 [5]. Most *Helicobacter* infections are transmitted where there is close contact, as between parent or sibling and infant. This accords with our finding that IP probands and their siblings share facets of the syndrome and increased prevalence of anti-urease antibody seropositivity [2]. Finding biological gradients between measures of IP, and their progression over 4 years, and a discriminant index for IP based on the serum immunoblot *H. pylori* antibody profile strengthens the case for causality. Increased frequency of clinically definite IP in urea-breath-test (UBT) positive spouses of probands [3] suggests that, in these circumstances, transmission of a primer can turn containment [6] into progression. *Helicobacter* emerged as a potential arbiter for progression.

In the natural history, brady/hypokinesia-predominant parkinsonism progresses to rigidity predominant [7]. Balance worsens, with increase in body sway, more reliance on visual correction and narrowing of ambulatory, coronal foot separation. If *Helicobacter* infection was the master switch in the pathogenic circuitry, its eradication should halt progression. Some recovery would be expected had it been a source of autoimmunity [1] or specific toxins [8], and microglial neurotrophic/homeostatic support was restored [9]. However, disease modification rather than global attenuation could result should a subsidiary pathogenic pathway be opened up.

We report on completion of the first study of the efficacy of *H. pylori* eradication on time-trends in the facets of IP, in probands receiving no, or only long elimination half-time (*t*½), anti-parkinsonian medication. An interim report [10] was made when the statistician was alerted to marked deterioration where eradication therapy failed. Persistence of *Helicobacter*, even at “low-density” (detected only by molecular microbiology on gastric biopsies) appeared detrimental. Similar outcomes of eradicating “low-density” and UBT-positive infection would further an autoimmune hypothesis. The history of a proband, whose biopsies were culture/histology negative but positive on polymerase chain reaction (PCR) assays, illustrates the case.

Understanding what is being measured, what influences it and exploring effect modification are keys to our approach [2]. All detected eradication failures [10] were anti-nuclear antibody (ANA) seropositive, as, indeed, are a quarter of probands [3]. ANA status is, thus, examined as an outcome-modifier in apparent successes. Similarly, the reduced blood lymphocyte count of IP [3] might be associated with impaired clearance of residual organisms.

## Methods

### Definition of Subjects

We have adopted Calne’s broad definition of “clinically definite” IP [11], rather than applying guidelines for the prediction of a single brain-pathology. The syndrome itself is the target, not the pathology. Realistically, different “neurodegenerative” pathologies occur in the same individual [12]. Between individuals, pathological response to the same environmental exposure may differ according to genetic predisposition and other modifying factors.

**Figure 1:**
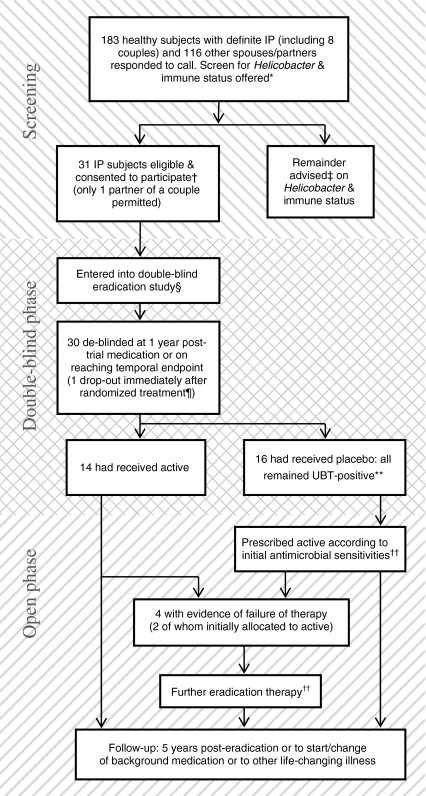
Study profile in probands with idiopathic parkinsonism (IP) and their spouses/partners. *For full analysis of screen, see [10]. †29/31 positive on all three screening tests: all were seropositive on immunoblot profile, one was urea-breath-test (UBT) negative, another had an equivocal anti-urease enzyme-linked immunosorbent assay (ELISA) value. Twenty-nine were culture positive for *Helicobacter pylori* (including the one UBT negative), remaining two (both UBT positive) were positive on PCR assay. *H. pylori*-like organisms on histology in 30, exception being culture positive. ‡With reference to further investigation and treatment. §Diarrhea during trial-medication (active) in one: compliance not affected and de-blinding as per schedule at 54 weeks. ¶No medical reason. **Any apparent spontaneous eradication was to be confirmed by endoscopic biopsy. ††Following open active, UBT offered at 6 weeks, with, if negative, a delay to endoscopy of at least 4 months.

The study was approved by local ethics committees, written consent obtained from all participants. Figure 1 shows the study profile. Responders to a call for volunteers were screened for *Helicobacter* (by UBT and serology) and immune status [10]. Accompanying spouses/partners were offered screening. Inclusion criteria were independently living probands with biopsy-proven *H. pylori* infection (on histology or culture). At endoscopy, “light” sedation (intravenous midazolam, maximum dose 4 mg) was an option.

Subjects receiving no anti-parkinsonian medication were preferred. Any “background” anti-parkinsonian medicine had to be long acting (making use of levodopa an exclusion), at steady state, and evenly spaced with reference to *t*½ to avoid iatrogenic fluctuations in performance. It must remain constant throughout.

Other exclusion criteria were: (1) secondary parkinsonism, “parkinsonism-plus” syndromes or other wider clinical entities [11,13]; (2) clinical depression (Beck’s Hopelessness score [14]), dementia (cognitive ability scales: Modified Tooting Bec score ≤8/16 and/or Mini-mental Score <24/30 [15,16]) or other mental illness; (3) other specific neurological condition; (4) cardiovascular/respiratory symptoms during normal activities; (5) other progressive or resolving disorders affecting physical ability or performance, or sufficient underlying incapacity to prevent assessments; (6) UK MRC muscle strength score <4/5; (7) concurrent therapy with potentially anti-dopaminergic drugs, or with hypnotics or sedatives; (8) recent change in life situation; (9) serious pathology, such as ulcer or neoplasm (any esophageal, gastric or duodenal lesion biopsied in line with standard practice) or inflammatory bowel disease.

### Protocol

This is a randomized, double-blind, placebo-controlled, parallel-group study of the effect of eradicating *H. pylori* on facets of parkinsonism. A computer generated randomization was applied to consented probands, numbered according to order of entry. De-blinding was scheduled for 54 weeks, or sooner if (1) current life-style threatened by disease progression (temporal end-point), (2) other exit criteria met (development of other medical conditions needing specialist attention; falls with serious injury/fracture; initiation of/modification to anti-parkinsonian medication), or (3) requested by subject. Judgments on premature de-blinding were made by two physicians, blind to randomization.

*Helicobacter* status was not re-investigated until after de-blinding. Probands who remained UBT positive after placebo were offered the corresponding active regimen. Those who received active had a UBT and were offered endoscopic biopsy. Follow-up was scheduled for 5 years post-eradication, but curtailed if any background medication was changed, new maintenance therapy started, or if another life-changing illness diagnosed.

Acquisition of objective measures of facets of parkinsonism and independent video analysis (blind to sequence/treatment) were used to minimize bias.

### Trial Medication

One group received 1 week’s triple therapy, the other matched-placebo capsules/tablets. Placebos were supplied by manufacturer, the only difference being the absence of active moiety. Medication was pre-packed according to patient number. The usual treatment was 20 mg omeprazole, 500 mg clarithromycin, and 1 g amoxicillin, 12-hourly. Twelve-hourly metronidazole 400 mg and/or 6-hourly tetracycline 500 mg were to be used where there was *in vitro* insensitivity and/or suspected intolerance. The appropriate three trial-medication containers were dispensed.

Where there was evidence that a blind- or open-active regimen failed, a different regimen was offered, taking into account any new anti-microbial sensitivities/inferences from PCR. Compliance was checked over the telephone.

### Assessment Schedule

Two baseline assessments, at a set time of day, preceded randomization. After the allocated treatment, assessments were scheduled 6-weekly. Follow-up after open active was as for blinded active. Where there was no evidence of continuing infection after an active treatment, follow-up was reduced, at 54 weeks, to 12-weekly for 1 year, and then to 24-weekly.

### Clinical Measurements

Cardinal outcome criteria were:

1. *Brady/hypokinesia of gait.* The primary outcome was hypokinesia, measured by mean stride length at free-walking speed, during “steady-state” [17], over 18 m in a 1.85 m wide corridor. Stride length usefully defines treatment effects and, when corrected for relevant demographic/anthropometric characteristics, discriminates well between those with and without diagnosed parkinsonism [18,19]. Rested subjects walked unaided, “at your own speed” [18]. A second walk followed 5 minutes rest. Stride length and speed were analyzed.
2. *Rigidity of upper limb.* To measure rigidity [20], the supported forearm was moved horizontally, at a controlled velocity, through a 40° arc about the elbow. The side judged more rigid at the initial assessment was studied. Swing duration was 1.3 seconds, the pause between varying (1–3 seconds) to reduce the effect of anticipation. One minute’s acclimatization preceded 2½ minute recording. A computer interface unit measured the torque required for passive displacement against position (sampling interval 25 ms). Mean torque for each swing was calculated, and the grand means taken for extension and for flexion. Greater rigidity in flexor muscles than in extensor is characteristic of parkinsonism.
3. *Tremor of hands.* Video recordings were used to assess tremor, as attaching devices to the hand can modify it. A fixed camera filmed a plan-view of hands, resting semi-prone on the table in front of the seated subject. It recorded any tremor over 1 minute before and during stress from repeating back, in reverse order, series of spoken random numbers [20]. The video archive was rated by an independent assessor, blind to date and treatment, using a visual-analog scale (from 0, the most intrusive tremor witnessed between-subject, to 100, no tremor), for each recording condition. Two others reviewed a sample (seven videos of one subject, whose unprovoked tremor ranged between extremes): their ratings did not differ significantly from the main assessor’s (mean difference 6 (95% CI −13, 25) and 3 (−7, 12), paired *t* tests).
4. *Postural abnormality.* Functional and anatomical abnormality contribute to imbalance in IP [7]. Total angular displacement (sway) in the sagittal plane was measured [7]: subjects stood at ease, with eyes open for 1 minute, then closed for three. Anatomical posture was gauged by height (with buttocks and heels against a wall, but otherwise standing relaxed) and, during walking, by mean coronal foot separation at mid-swing [7,18].

Complementary criteria were:

*Stance/walk videos.* A “blinded” independent assessor analyzed the entire archive of anterior and lateral videos of standing (15 seconds) followed by walking (over 12.5 and 6.5 m, respectively). Lateral perspective was from the side on which rigidity measured. Another assessor reviewed a sample (total 153 anterior and 128 lateral videos, from 20 subjects). On a global rating visual-analog scale (0 mm representing a subject’s worst walk, 100 his/her best), there was no significant difference in their ratings (mean difference −1.8 (−6.5, 2.9) for anterior, 1.0 (−4.5, 6.5) lateral).
Scales were constructed to describe brady/hypokinesia and tremor (from 0, most severe abnormality witnessed between-subject, to 100, normality). The brady/hypokinesia scale took equal account of 12 items, each formulated from correlated observations (e.g. arm swing on more-rigid and less-rigid sides during walk). Each observation contributed equally to an item. The tremor scale (more-rigid side only) took equal account of three observations (during walk from anterior and lateral perspectives, and at stance from lateral). Chronbach’s alpha [21] provides an estimate of within-scale consistency. It was 0.84 and 0.91 for 12-item brady/hypohypokinesia and 3-observation tremor scale, respectively, indicating satisfactory scales.
*Psychometric disability.* Reaction time was measured as that taken to lift left or right index-finger from its touch-sensitive support [22]. Two seconds before the imperative, an alerting signal did, or did not, warn whether left or right was to be lifted. “Cognitive inefficiency” was measured by the ratio, warned/unwarned.
*Other measurements.* Rested supine, standing (immediate and at 1 and 3 minutes) and post-exercise blood pressure and pulse were measured, as was body weight (unshod, without outer clothing).

### Histologic, Microbiologic and Immunologic Methods

Infection was proven by: (1) gastritis associated with *Helicobacter*-like organisms on microscopy (any of six biopsies: anterior and posterior antral, incisurial, anterior and posterior corporal, cardiac), and/or (2) culture of *H. pylori* (either of two biopsies: antral and corporal, couriered to the *Helicobacter* Reference Laboratory), or, if culture negative, (3) detection of *H. pylori-*specific DNA. Biopsies, ordered on a cellulose acetate strip, were fixed (10% buffered-formalin) and processed to paraffin blocks. Hematoxylin and eosin stained sections were examined and classified (Updated Sydney System [23]), organisms sought using cresyl-fast violet. The *Helicobacter* Reference Laboratory operating procedures for *H. pylori* culture were used, with in vitro testing for susceptibility, to clarithromycin, amoxicillin, metronidazole and tetracycline, by Etest (AB Biodisk, Solna, Sweden) [24]. Culture negative biopsies were tested using a PCR targeting 16S rRNA (primer pair HP1/HP2 [25]) and *vacA* (Vac3624F/Vac3853R [26]) genes: sensitivities on gastric biopsies were 90 and 85%, respectively, specificities 99 and 98% [26]. Where positive, clarithromycin susceptibility was determined using real-time PCR [26].

In the screen, *Helicobacter* status was defined by [^13^C]urea-breath-test (INFAI, Kestrel Healthcare Ltd., Basingstoke, UK) and serology. Serum was stored at −20 °C. An enzyme-linked immunosorbent assay (ELISA) for anti-urease-IgG antibody (SIA417A; Delta Biologicals, Rome, Italy) had between-assay coefficients of variation, for the samples assayed in singlicate, of 5.8 and 8.1% at an ELISA value of 1.7 and 3.2. An ELISA value >2.2 is the recommended cut-point of seropositivity, from equivocal/seronegative (1.8 to 2.2/<1.8). Presence/absence of defined antibody bands on Western blotting was scored according to the manufacturer’s system (RIDA *Helicobacter* Blot IgG; Quadratech Diagnostics Ltd., Epsom, UK). Blots and the developed control (k) strip, supplied with each kit, were scanned and magnified, to maximize precision of band detection. A score >12 is the recommended cut-point of positivity, from equivocal/negative (11 to 12/<11). Each assay kit contains a negative and a positive quality control: all read true.

Serum was screened for ANA by indirect immunofluorescence using Hep2010 cells and rat stomach, kidney and liver composite tissue block (Biochip slides; Euroimmun UK, Pontypool, UK). Secondary antibodies were FITC-conjugated polyclonal rabbit anti-human IgG (Dako, Ely, UK). Lymphocyte subset counts were obtained by flow cytometry: four-color fluorescent cell labeling using the MultiTEST kit in TruCOUNT tubes and a FACSCalibur flow cytometer (Becton Dickinson, San Jose, CA, USA). These standard immunological procedures were subject to the UK national external quality assurance scheme.

### Statistical Methods

Sample size calculation was based on the primary outcome criterion, stride length. It was estimated that, for a significance level of .05 and a power of .8, 56 subjects (28 in each group) would be required to show a difference between active and placebo treatments of ¾(SD) at 1 year, where SD is the between-subject standard deviation.

When the statistician was alerted to an obvious difference in clinical progression where blinded active eradication therapy failed [10], 30 subjects had commenced the post-treatment assessments, 20 had reached de-blinding. In this 20, an intention-to-treat analysis on the final measurement of stride length in the blinded-phase showed a statistically significant difference in favor of active treatment (*p* = .02). The null-hypothesis was rejected: “a *p*-value of .05 from a small sample can be quite strong evidence, but one of .05 from a larger sample is always weak” [27]. Steering and ethical committees were informed, recruitment halted. All 30 entered were to continue to and beyond de-blinding.

Detailing drop-outs and outcome of treatment failure are integral to any efficacy study. In the double-blind protocol analysis, nature of treatment refers to “successful” active (no evidence of failure) or to placebo. Linear mixed models [28] were used to analyze repeated measures of primary, other cardinal and complementary outcome criteria. Fixed effects in the model were: nature of treatment; time since treatment; any background anti-parkinsonian medication; their interactions (between: treatment and time, background-medication status and time, treatment and background-medication status); mean baseline measurement; and, where relevant, demographic /anthropometric characteristics. A random effect for subject was included in the model: thus, it contained both a subject-level random intercept and the usual residual term, each assumed to have a normal distribution. Fixed effects were kept in the model regardless of whether they reached conventional levels of statistical significance. Models were fitted via the *xt* commands within stata 10 [29]. After visual inspection for broad similarity and linearity of individual subjects’ temporal trends, the efficacy of eradication was assessed via the interaction between nature of, and time since, treatment. Estimated annual change in an outcome was obtained directly, or by linear combination of model coefficients, according to nature of treatment.

Variables exhibiting a positively skewed distribution were transformed (natural logarithmic) to approximate normality.

### Case History of Eradication Therapy for Low-Density *H. pylori* in Idiopathic Parkinsonism

This patient with untreated IP (5 years from diagnosis) provided the first opportunity to examine prospectively the effect of eradicating low-density *H. pylori* infection. Had she presented earlier, she would not have been eligible for the efficacy study, where all recruited had frank *Helicobacter* infection (UBT and/or culture positive). Her spouse was UBT positive, and her mother had recently been treated for *Helicobacter.* Her sister and late father “always had stomach problems,” the latter having been treated for peptic ulcer. There was no family history of diagnosed parkinsonism, but her mother had a tremor. She had a 2-year history of acid regurgitation and “needing food between meals,” and felt bloated and nauseated on rising and after eating. She complained of nocturnal hot-sweats. She has never smoked. Table 1 gives further baseline characteristics, including gastric biopsy results. The likelihood of a biopsy giving a false positive with multiple PCR primer pairs is small [34].

**Table 1:** Baseline characteristics of patient with low-density *Helicobacter pylori*

Characteristic	On presentation
Personal	Female, aged 59 years, weight 84 kg, height 1.6 m, body mass index 32.8 kg/m^2^
Manifestations	Brady/hypokinesia-predominant idiopathic parkinsonism, untreated
Blood profile	Full bood count, B-lymphocyte and T subset counts: normal. Serum vitamin B12, folate and ferritin: normal. Homocysteine = 21 μmol/L (target <16). Autoantibody screen: anti-intrinsic factor positive, otherwise negative including anti-nuclear antibody
*Helicobacter* screen	Urea-breath-test negative: immunoblot score positive: anti-urease ELISA not done
Gastric biopsy sites	Three sets (antral and corporal): two sets for histology, one microbiology
Histology	Mild chronic antral/corporal inflammation, no polymorphonuclear activity or *Helicobacter*-like organismsseen. No atrophy or intestinal metaplasia
Culture	Negative
Molecular microbiology^a^	Positive for *Helicobacter* DNA from three different *vacA* regions (s1, m2, and intermediate) in antrum, for mid-region (m2) only in corpus; both biopsies *cagA* negative

a Genomic DNA extracted from biopsies (Qiagen DNeasy Tissue kit, Qiagen Ltd, Crawley, UK) and the presence of *H. pylori* genomic DNA tested by PCR amplification.Primers specific for three regions of *vacA* gene used: (1) signal-region (primers VA1-F and VA1-R [30]); (2) mid-region (primers VAG-F and VAG-R [31]); (3) intermediate-region between signal and mid (primers DL2 and VacR9 (see [32] for primer sequence)).

Signal- and mid-region primers determine *vacA* type by product size and hence are expected to give a product for all *H. pylori* strains. The additional primer pair DL2/VacR9 amplify the entire intermediate-region, and are not specific for i-region type, hence are also expected to give a product for all *H. pylori* strains. PCR product sizes were estimated by agarose gel electrophoresis with reference to positive control strains and a commercial DNA ladder. Product for signal-region reaction matched size of that for s1 control strain 60190 (259 bp); mid-region product matched that obtained for m2-type control strain Tx30a (645 bp). Intermediate-region amplification gave a band similar to that of 60190 (361 bp), the expected size for primer used. Negative control samples (no template) demonstrated that contamination of reagents with *H. pylori* DNA (a likely reason for a false-positive result) had not occurred.

The s1 signal-region allele associated with in vitro vacuolating activity, s2 Vac protein being nonvacuolating. Mid-region affects epithelial cell binding, m1 toxin vacuolating a wider variety of cells [32]. Thus, s1/m1 strains are vacuolating, s2/m2 nonvacuolating and s1/m2 variable. Intermediate-region interacts in vacuolation, according to subtype (not determined). Decreased propensity toward gastric disease expected with *cagA* negativity.

Primers for *cagA* were cag2 and cag4 [33].

Following two baseline clinical assessments, she received a 1-week anti-*Helicobacter* course (12-hourly ranitidine bismuth citrate 400 mg, amoxicillin 1 g, and clarithromycin 500 mg). Her spouse was treated contemporaneously. Her assessments were repeated approximately 2-monthly over the next 3.4 years. Biopsies taken 15 months post-treatment showed no evidence of *Helicobacter* (same protocol/team): the serum immunoblot score had become negative by 2.5 years. Hydrogen-breath-tests for small intestinal bacterial overgrowth (SIBO) were carried out, using a 25 g lactulose test dose, following 24-hour deprivation of dairy products (and any medicinal lactulose) and a breakfast of 250 mL black tea/coffee or water. Hydrogen concentrations were measured (Micro Medical Ltd., Rochester, UK) pre-dose and at 15-minute intervals for 4 hours after. Criterion for positivity was two consecutive values [35] ≥cut-point of meter manufacturer. A generalized estimating equation model, with first order autoregressive correlation structure, was used to estimate the average performance in four time-periods, relating to *Helicobacter* status and hydrogen-breath-test status.

## Results

### Intention-to-Treat Analysis

Table 2 gives baseline clinical measurements and pre-treatment histopathology/microbiology, according to randomization: there was no clinically important imbalance in measurements between the two groups.

**Table 2:** Pre-randomization characteristics of efficacy-study subjects

	Mean (data interval)	
Characteristics	Placebo (n = 16)	Active (n = 14)
Demographic/anthropometric		
Age (years)	63 (45, 81)	59 (41, 78)
Gender^a^	13 M, 3 F	6 M, 8 F
Height (m)	1.72 (1.57, 1.88)	1.69 (1.48, 1.89)
Weight (kg)	80.9 (53.8, 108.2)	77.9 (50.5, 105.2)
Mini-mental score (maximum 30)^b^	28 (27, 29.5)	29 (29, 30)
Modified Tooting Bec score (16)^b^	15.25 (15, 16)	15.75 (15, 16)
Beck’s Hopelessness score (20)^b^	3 (1, 5)	3.5 (2, 6)
Background anti-parkinsonian medication (no/yes)	8/8	9/5
Brady/hypokinesia		
Mean stride length (mm)	1244 (909, 1579)	1222 (806, 1638)
Free-walking speed (m/second)	1.20 (0.79, 1.60)	1.14 (0.64, 1.65)
Rigidity		
Mean torque to extend forearm (Nm × 10^−3^)^c^	400 (156, 1026)	463 (160, 1335)
Mean torque to flex (Nm × 10^−3^)^c^	290 (111, 756)	326 (165, 643)
Tremor rating (100 none, 0 worst) global scale		
Mean tremor seated rest^b^	96.7 (83.4, 100)	92.2 (69.5, 97.5)
Mean tremor seated, stress^b^	88.8 (74.3, 100)	88.0 (62.0, 98.5)
Mean tremor during stance/walk^b^	92.3 (89.3, 100)	95.4 (96.9, 100)
Postural abnormality		
Mean body sway, eyes open^°C^	5.6 (2.2, 14.4)	5.1 (2.2, 11.5)
Mean body sway, eyes closed^°C^	9.1 (3.1, 27.1)	8.2 (2.8, 24.1)
Mean foot separation (mm)	198 (149, 247)	194 (144, 244)
Psychomotor and psychometric measures		
Mean unwarned reaction time (ms)^c^	608 (408, 907)	566 (369, 868)
Mean warned reaction time (ms)^c^	366 (206, 653)	347 (197, 611)
Histopathology and microbiology, gastric biopsies^a^		
*Helicobacter*-like organisms on histology^d^	15	14
Culture +ve	16	13^e^
Metronidazole resistance, antral biopsy^f^	6 (1)	5 (2)
Metronidazole resistance, corporal^f^	6 (1)	3 (2)
Immunoblot +ve for CagA antibody	13	13

Inspection shows no evidence of a pre-randomization characteristic (except gender) being associated, by chance, with a randomization category. In the analysis, outcome measures are corrected for personal characteristics (including gender), where pre-determined to be appropriate.

a Count.

b Median (interquartile range).

c Exponential of log_e_ transformation (see Statistical Methods).

d Pangastritis (n = 12), antral predominant (17), corpus predominant (1). Chronic inflammatory activity (grading normal as 0, mild 1, moderate 2, marked 3) not significantly different for active and placebo randomizations in antrum (median (interquartile range) 2(2, 2), 2(1, 2), respectively) or corpus 1(0, 2), 1(1, 1), nor was polymorphonuclear activity (antrum: 1(1, 1), 1(1, 2); corpus: 1(0, 1), 1(0, 1)) or *Helicobacter pylori* density (antrum: 2(2, 2), 2(2, 3); corpus: 1(1, 2), 1(1, 2)). Atrophy (n = 5 (marked in 1, moderate 2, mild 2); intestinal metaplasia in 4 of these (marked 2, mild 2)) not associated with randomisation category.

e In remaining subject, PCR for *H. pylori* (*vacA*) positive on both biopsies, clarithromycin mutation assay showing wild (sensitive) type.

f Number with metronidazole resistance (intermediate susceptibility). All cultures sensitive to clarithromycin, amoxicillin, and tetracycline. Second-line anti-microbial regimen prescribed in four subjects (blinded active randomization) because of history of intolerance: Rx clarithyromycin with metronidazole in three, with tetracycline in one.

An intention-to-treat analysis on the final measurement of the primary outcome, stride length, in the blinded-phase (n = 14 for active, n = 16 placebo) gave a *p-*value of .005 (adjusted for baseline stride length (*p*< .001), age, height, and gender [19] not contributing to variance explained). Rejection of the null-hypothesis was confirmed, a multiplicity adjustment not considered necessary. The magnitude of effect was equivalent to 1.02 times the between-subject standard deviation: the study had been designed to detect 0.75(SD) with a sample size of 56. The corresponding time-trend in stride length was 69 mm/year better after blinded active than after placebo (active 59 (95% CI 26, 92); placebo −10 (−46, 25)).

Given concern about safety, an intention-to-treat analysis was also performed on the final measurement in torque to extend the forearm. It gave a *p*-value of .006 (adjusted for baseline (*p* = .001), there being no pre-identified personal covariate [20]). The magnitude of effect was only 0.11 (SD). The corresponding time-trend in torque was 205 Nm × 10^−3^/year worse after blinded active than after placebo (247 (136, 359); 42 (−55, 139), respectively). Torque to flex was unaffected.

### Protocol Analysis

Table 3 gives the double-blind protocol analysis of time-trends in clinical measurements (two proven eradication failures of blinded active excluded). Numbers reaching a temporal end-point and duration of follow-up are given. Table 3 also compares outcomes in the subsequent year, between probands initially allocated to active and those receiving open active after placebo (two failures of open active excluded).

**Table 3:** Effect of blinded- and open-*Helicobacter* eradication on within-subject time-trends in outcome criteria for idiopathic parkinsonism

	Estimated change in blinded phase^a^^b^Mean (95% CI)/year			Estimated change subsequent year^a^ Mean (95% CI)/year		
Outcome variable	Placebo 86 assessments (n = 16)	Active 82 assessments (n = 12)^c^	*p*-value	Open active 81 assessments (n = 14)^c^	Post-blinded active30 assessments (n = 12)	*p-*value
Brady/hypokinesia^d^						
Stride length (mm)	3 (−47, 52)	75 (41, 110)	.01	69 (2, 136)	−26 (−121, 70)	.08
Free-walking speed (mm/second)	86 (1, 174)	92 (30, 153)	.9	83 (2, 163)	−27 (−148, 94)	.1
Rigidity (Nm × 10^−3^)						
Torque to extend	−1 (−141, 143)	238 (115, 361)	.01	197 (49, 344)	92 (−94, 278)	.4
Torque to flex	1 (−92, 94)	59 (−214, 140)	.3	47 (−105, 200)	−23 (−141, 95)	.4
Tremor rating (100 none, 0 worst) global scale						
Tremor seated^e^	−3 (−11, 4)	0 (−5, 6)	.4	−11 (−19, −2)	−1 (−13, 11)	.2
Tremor stance/walk	−5 (−12, 3)	4 (−2, 9)	.07^f^	0 (−9, 8)	−13 (−26, 1)	.1
Postural abnormality (%)^g^						
Body sway	−2 (−8, 4)	3 (−2, 8)	.2	−1 (−7, 5)	−2 (−12, 8)	.8
Ratio sway: eyes open/closed	2 (−6, 10)	2 (−5, 9)	.9	5 (−4, 14)	−3 (−16, 11)	.3

a Median follow-up before deblinding: 263 (inter-quartile range 103–378) days following placebo (n = 16, with premature deblinding in 9), and 352 (261–370) following successful blinded active treatment (n = 12, with premature deblinding in 3). There was evidence of eradication failure in two probands receiving blinded active, and two receiving open active.

b Following unaffected in protocol analysis: psychometric function, mean arterial pressure, pulse and body weight.

c Parameter estimates were similar, when failure of eradication was used as an effect-modifier in whole group, to estimates where failures were simply excluded.

d No significant effect of eradication therapy on subjective brady/hypokinesia video rating.

e No significant effect of eradication therapy on increment in tremor under stress.

f After excluding those with tremor ratings of ≥90/100 on stance/walk, size effect: −18 (95% CI −42, 7)/year after placebo (n = 6, 29 observations), 8 (−7, 24) after blinded active (n = 4, 29 observations), *p* = .06.

g No significant effect of eradication therapy on ambulatory foot separation.

Stride length improved (by, on average, 73 mm/year) following successful blinded active compared with placebo. Free-walking speed was unaffected by the double-blind treatment, but optimizing stride length would have made gait more energy-efficient.

**Figure 2:**
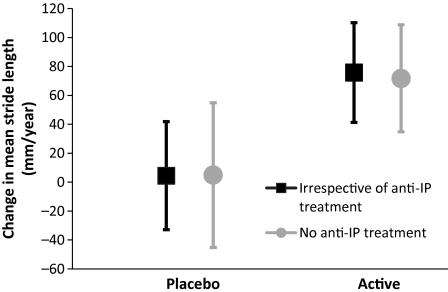
Comparison of time-trend in stride length, following double-blind successful-active *Helicobacter pylori* eradication therapy and placebo, between entire group (n = 28) and those not exposed to background anti-parkinsonian (IP) medication (n = 17). Estimated change (95% CI) over a year is shown. After excluding those on anti-parkinsonian medication, difference between treatments remained significant (*p* = .035), size of effect similar (placebo: 5 (−45, 55) mm/year (n = 8); active: 72 (35, 109) (n = 9)). Two probands, in whom blinded active treatment failed, are excluded: they and 11 others were receiving background medication.

Excluding those receiving background anti-parkinsonian medication did not diminish the size of treatment-effect on stride length (Fig. 2). That is the effect of eradicating *Helicobacter* was not attributable to improved absorption of, or response to, anti-parkinsonian medication.

**Figure 3:**
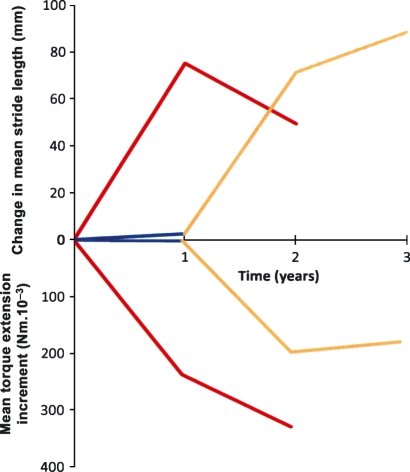
Schematic representation of effect of eradicating *Helicobacter*, on hypokinesia and rigidity, over 2 years. Estimated mean time-trends in stride length and torque to extend forearm following successful blinded active (red, n = 12) and placebo (blue, n = 16) are shown. Change in outcome measure is also given for year 2 after blinded active (red, n = 12), and for each of the 2 years following successful open active (orange, n = 14). Time-trends for the year after blinded active or open active were significant (stride length: *p* = .001 and .04, respectively; torque: *p* = .001 and .009). Time-trends after placebo and in year 2 after blinded active were not (Table 3), neither were those in the second year after open active (n = 8 with 48 assessments). There was no significant time-trend in stride length (n = 9 with 40 assessments) in year 3 after blinded active (or even on pooling year 2 and 3 data). However, a significant increment in torque (88 (95% CI 15, 161) Nm × 10^−3^, *p* = .02) occurred in year 3.

The statistically significant effect on stride length of successful open active in year 2 is illustrated in Fig. 3. Its magnitude, compared with placebo, was similar to that after blinded active. There was no further significant change in the second year after blinded active or open active. Moreover, there was no evidence of deterioration in the third year after blinded active.

Mean torque required to extend the forearm was greater (by 239 Nm × 10^−3^/year, i.e. 52% of baseline) following successful blinded active than following placebo (Table 3), irrespective of anti-parkinsonian medication. Successful open active had a statistically significant effect on torque to extend, approaching the same magnitude relative to placebo (Fig. 3). There was no significant time-trend in the second year after an active treatment. A small deterioration in the third year after blinded active was insufficient to affect stride. Torque to flex the forearm had a lower baseline than that to extend (Table 2): the characteristic parkinsonian rigidity pattern. It was unaffected by treatment (Table 3).

Thus, two facets of parkinsonism dissociate after eradicating *Helicobacter*, with change in stride and rigidity in mirror image (Fig. 3).

Tremor during stance/walk tended to increase after placebo relative to blinded active, the size of effect being large (−26/year in the 10 probands with tremor ratings ≥90/100, where 100 is no tremor, 0 most intrusive). Increase in torque to extend after blinded active may have dampened tremor. Tremor while seated was not sensitive to the treatment-effect. Anxiety at the hands being under scrutiny may have dominated.

Successful *Helicobacter* eradication had no effect on postural measures, cognitive efficiency, mean arterial pressure and pulse, or body weight.

### Failed Eradication

Table 4 gives the detailed analysis of time-trends in four probands with evidence of failed eradication, compared with the first year after successful treatment in the remaining 26. All failures were receiving background anti-parkinsonian medicine. Their cytotoxic T-cell (CD8+) count was lower by 323 (95% CI 50, 596)/μL (adjusted for age and gender [3], *p* = .02). (Median 526 (interquartile range 390, 641)/μL in remainder, corrected to mean age 60 year and as if all subjects male). Total white cell, neutrophil, lymphocyte, T helper (CD4+), B cell (CD19+) and natural killer (CD16+56+) counts did not differ. All failures, but only 6 of the remainder, were ANA positive (Fisher’s exact test, *p* = .008).

**Table 4:** Natural experiment of eradication failure, and effect of anti-nuclear antibody (ANA) status, on within-subject time-trends in outcome criteria for idiopathic parkinsonism, in first year post-active treatment

	Estimated change/year^a^^b^Mean (95% CI)			Estimated change/year^a^^b^Mean (95% CI)		
Outcome variable	Evidence failed eradication^c^22 assessments(n = 4)	Remainder 153 assessments (n = 26)	*p-*value	Remainder ANA +ve 25 assessments (n = 6)	Remainder ANA −ve 128 assessments (n = 20)	*p-*value
Brady/hypokinesia^d^						
Mean stride length (mm)	−158 (−273, −43)	52 (17, 87)	.001	−105 (−196, −14)	68 (33, 102)	.001
Mean free-walking speed (mm/second)	−97 (−271, 76)	72 (19, 124)	.046	−64 (−206, 79)	85 (31, 139)	.045
Rigidity (Nm × 10^−3^)						
Mean torque to extend	374 (76, 672)	160 (61, 258)	.1	−113 (−346, 121)	191 (92, 291)	.01
Mean torque to flex	−41 (−283, 201)	−2 (−83, 80)	.7	−140 (−333, 53)	16 (−68, 101)	.1
Tremor						
Mean tremor seated (mm)^e^	−8 (−24, 8)	−3 (−8, 2)	.7	5 (−8, 18)	−4 (−8, 2)	.2
Mean tremor stance/walk (%)	4 (−11, 19)	2 (−3, 7)	.7	−15 (−26, −3)	4 (−1, 8)	.003^f^
Postural abnormality (%)^g^						
Mean body sway	0 (−42, 78)	5 (−12, 24)	.9	−9 (−46, 44)	6 (−11, 26)	.5
Ratio mean sway: eyes open/closed	−12 (−28, 8)	−1 (−7, 6)	.2	−16 (−29, 0)	1 (−5, 8)	.04

a Median follow-up after known eradication failure was 268 (range 78–416) days, and, in remainder 544 (inter-quartile range 245, 1059).

b Psychometric function was unaffected by either eradication failure or ANA status. Mean arterial pressure was unaffected, but pulse lower with eradication failure (−11 (95% CI −20, −1) vs 1 (0, 3) supine; −9 (−19, 1) vs 1 (0, 3)/minute/year standing over 3 minutes: *p* = .01 and .048 respectively). Weight loss was greater with failure (−0.3 (−1.3, 0.1) vs 0.6 (−0.2, 1.4) kg, *p* = .03). ANA status had no significant effect on these outcomes in remainder.

c Criterion: UBT (n = 1), histology and culture (1), molecular detection only (2). UBT available in all 30 following an active treatment, 26/30 having a repeat endoscopy at a median of 346 (inter-quartile range 31, 387) days. Classification and grading of gastritis improved (even in those with evidence of eradication failure) or returned to normal, in all but one. The exception had a minor degree of chronic antral gastritis, with *Helicobacter*-like organisms before eradication, without organisms after.

d Subjective brady/hypokinesia video rating deteriorated with eradication failure (−13 (95% CI −25, −0.3) vs 1 (−3, 5), *p* = .02). It was unaffected by ANA status in remainder.

e Tremor under stress unaffected by eradication failure or ANA status.

f After excluding those with tremor ratings of ≥90/100 on stance/walk, size effect: −40 (−74, −6) for ANA positive (n = 2 with eight observations), 7 (−5, 20) for ANA negative (n = 7 with 48 observations), *p* = .004.

g Ambulatory foot separation tended to be narrower with eradication failure (−28 (−51, −4) vs −7 (−14, 0.5) mm, *p* = .07), ANA status having no significant effect in remainder.

Stride length was worse by, on average, 210 mm/year with eradication failure compared with the remainder, with a concomitant relative decline in free-walking speed (169 mm/second/year). Subjective assessment of videos was able to detect a difference of this magnitude (−14/year, where 100 is normal, 0 severe brady/hypokinesia).

Torque to extend the forearm was numerically greater with eradication failure, but the difference from the remainder reached significance only at the .1 level. Torque to flex was unaffected.

Tremor, body sway, and standing height were unaffected, but foot separation tended to be narrower (21 mm) with failure. It had no effect on cognitive efficiency or blood pressure. With failure, pulse rate was lower by 12/minute/year lying and by 10 standing, the effect being masked by exercise. Weight loss with failure was clinically unimportant.

### Anti-Nuclear Antibody Status as an Effect-Modifier in “Successful” Eradication

**Figure 4:**
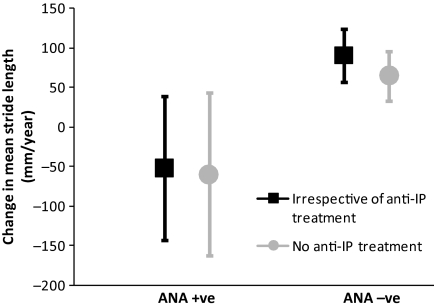
Comparison of time-trend in stride length, in anti-nuclear antibody (ANA) positive and negative, between the entire group with “successful”*Helicobacter pylori* eradication (n = 26) and those not exposed to background anti-parkinsonian (IP) medication (n = 17). Estimated change (95% CI) over a year is shown. After excluding those on anti-parkinsonian medication, difference with ANA status remained significant (*p* = .02), size of effect similar (ANA positive: −60 (−162, 43) mm/year (n = 4); ANA negative: 65 (34, 96) (n = 13)). Four probands in whom active treatment failed are excluded: they and six of the remainder were ANA positive.

Excluding known eradication failures, stride length declined in the ANA positive compared with the ANA negative, by, on average, 173 mm in the first year after anti-*Helicobacter* therapy (Table 4). There was a concomitant relative decline in free-walking speed (149 mm/second/year). The effect was not confounded by background anti-parkinsonian medication (Fig. 4).

Torque to extend the forearm was less by 304 Nm × 10^−3^/year in the ANA positive (Table 4), irrespective of anti-parkinsonian medication, as though positivity “protects” against the increase in rigidity expected with successful eradication. Torque to flex also showed a numerically greater decline (156 Nm × 10^−3^/year) in the ANA positive, but the difference reached significance only at the .1 level.

Tremor during stance/walk worsened in the ANA positive (47/year in those with tremor ratings ≥90/100) relative to the ANA negative, consistent with relative decrease in parkinsonian rigidity with positivity. Tremor while seated was unaffected.

Provocation of imbalance by closing the eyes was greater (17%/year) in the ANA positive, but standing sway overall, height and ambulatory foot separation were unaffected. ANA status had no effect on cognitive efficiency, mean arterial pressure, pulse or body weight.

Anti-nuclear antibody status was not associated with the presence/absence of antibody against cytotoxicity-associated gene product (CagA) on the immunoblot. This was addressed because anti-CagA-seropositivity is associated with greater deterioration in parkinsonian facets with time [36].

### Eradication of Low-Density *H. pylori* Infection: Case History

Acid regurgitation and bloating with nausea resolved following *Helicobacter* eradication (possibly due to improved gastric emptying [37]), hot-sweats disappeared.

**Figure 5:**
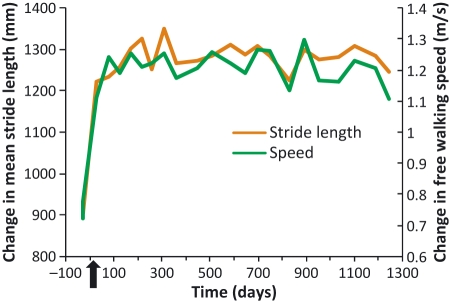
Mean stride length and free-walking speed following biopsy-proven eradication of “low-density”*Helicobacter pylori* infection in a patient with untreated idiopathic parkinsonism. After two baseline assessments, on separate occasions, the patient received a 1 week *Helicobacter* eradication course. Arrow indicates its completion.

There was marked sustained improvement in stride length and free-walking speed from the first assessment following eradication, at 6 weeks, until the last assessment 3.4 years after (Fig. 5). Stride length improved by 424 (95% CI 379–468) mm for 15 months post-eradication (*p* = .001), and correction of the deficit continued. She remained free from anti-parkinsonian medication throughout.

Following the baseline (phase 1), serial hydrogen-breath-tests remained negative (3/3) over 450 days post-eradication (phase 2). They were positive (6/7) over the next 500 days (phase 3). Over the 300 subsequent days (phase 4), when bulk and osmotic laxatives were exhibited and fluid consumption increased, tests were negative (3/3). The interposing period of hydrogen-breath-test positivity did not influence stride length or free-walking speed. Subjective assessment of videos easily detected the improvement of brady/hypokinesia, in phases 2, 3, and 4. Over baseline (*p* = .001, in each case).

**Figure 6:**
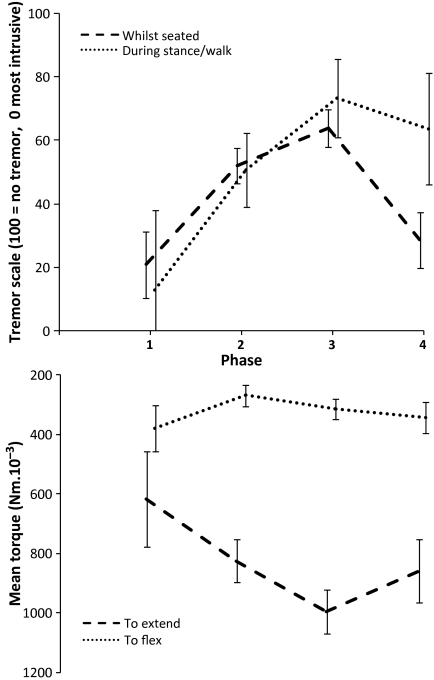
Mean (95% CI) rigidity and tremor before (phase 1) and after (phase 2) biopsy-proven eradication of “low-density”*Helicobacter pylori* infection, and before (phase 2), during (phase 3) and after (phase 4) hydrogen-breath-test positivity, in case history. Lower graph: Torque to extend forearm was less in phase 1 than subsequent phases (*p* = .02, .001, .01, respectively), but greater in phase 3 than either phase 2 or phase 4 (*p* = .001 and .04). Torque to flex was less than that to extend throughout. It changed little between phases (less in phase 2 than previously (*p* = .01), with a subsequent slow return toward baseline). Upper graph: Tremor whilst seated and during stance/walk showed similar patterns. Whilst seated, it was less intrusive in phases 2 and 3 than at baseline (*p* = .03 and .003), but deteriorated in phase 4 compared with phase 3 (*p* = .001). Stance/walking tremor was better than at baseline in all subsequent phases (*p* = .001, in each case), but did tend to deteriorate in phase 4 compared with phase 3 (*p* = .08).

Figure 6 (lower) shows that torque to extend the forearm was greater in phases 2–4 than at baseline. It was greater during the period of hydrogen-breath-test positivity (phase 3) than in preceding and succeeding periods of negativity. Torque to flex was lower than that to extend, and relatively unchanged throughout. There was a small (average 1 cm), but significant, loss of height in phases 2–4 (*p* = .001, in each case), compatible with a more flexed posture. Increase in torque to extend was accompanied by reduction in tremor, decrease by worsening of tremor (Fig. 6, upper).

Body sway decreased following *Helicobacter* eradication, and dependence of balance on vision was consistently less (Table 5). There was a concomitant increase in ambulatory foot separation. Sustaining a walk over 18 m had been difficult at baseline. Improvement in balance was also evidenced by the patient cycling 10 miles. Although there was a statistically significant fall in mean arterial pressure, supine (by 8 mmHg) and post-exercise (13 mmHg), the latter evidently did not affect balance adversely (Table 5). Her 5.2 kg weight loss should reduce sway, have no effect on reliance on vision and make foot separation narrower. (Weight is a covariate for sway and foot separation [7]). Weight loss may be due, in part, to decreased stasis edema.

**Table 5:** Balance before (phase 1) and after (2) *Helicobacter* eradication, and before (2), during (3) and after (4) hydrogen-breath-test positivity, in case history

	Mean (95% CI)			
Characteristic^a^	Phase 1	Phase 2	Phase 3	Phase 4
Mean body sway°^b^	14 (11, 17),	9 (8, 10)	9 (8, 11)	7 (6, 9)
Mean ratio sway: eyes open/closed (%)^c^	27 (21, 36)	74 (66, 84)	67 (59, 76)	51 (41, 64)
Mean foot separation (mm)^c^	168 (160, 175)	195 (191, 200)	193 (188, 197)	192 (186, 199)

a Supine mean arterial pressure numerically lower in successive phases, difference from baseline significant (*p* = .046) at phase 4 (mean 102 (95% CI 95, 108) and 94 (90, 98) mmHg for phases 1 and 4, respectively). Similar trend in standing pressure did not reach statistical significance, but progressive fall in post-exercise pressure did (112 (105, 119) and 99 (94, 104) for phases 1 and 4, *p* = .003). Supine pulse increased significantly (*p* = .009) over baseline by phase 4 (69 (64, 74) and 78 (74, 81)/minute for phases 1 and 4), standing tended to, but post-exercise pulse unchanged.

Body weight progressively decreased from baseline (81.9 (78.9, 84.9), 79.3 (76.8, 81.7), 77.3 (74.8, 79.7) and 76.7 (73.9, 79.6) kg for phases 1–4 respectively, *p* = .04, .005, and .007).

b For comparison of phases 2, 3, and 4 with phase 1, *p* = .005, .007, and .001 respectively.

c For comparison of phases 2, 3, and 4 with phase 1, *p* = .001 in each case.

There was no change in cognitive efficiency between phases. However, mean reaction time was significantly (*p* = .02) faster during hydrogen-breath-test positivity (phase 3) than at baseline (572 (95% CI 496, 659), 529 (497, 562), 477 (467, 509) and 493 (449, 543) ms for phases 1–4, respectively).

## Discussion

### Towards a Paradigm Shift

We question the classification of IP as non-communicable, and the corollary that any constitutional illness in probands has a separate cause. A whole template for ameliorating or halting progression is set aside by ignoring IP’s systemic nature [2,3], which includes morphological/neurochemical changes in the enteric nervous system [38,39], and prodromal slow gastrointestinal-transit [40,41] and peptic ulceration [4]. Moreover, Lewy bodies (a classical hallmark of Parkinson’s disease) and peripheral inflammatory cells have been reported in dopamine-cell brain implants [42]. The simplest explanation is that the recipient’s ongoing inflammatory process affects the transplant. A more fundamental nosological grouping could position IP at an interface of disciplines with potential for intervention in the pathogenesis. However, prevailing paradigm bias is imposing lines of demarcation which are time and criticism dependent [43].

Presentation and evolution of IP is diverse. Clinicians strive to split-off homogenous subsets, presumably to reveal that necessary and sufficient cause. We reject this approach, taking a broad clinical definition and quantifying individual facets and associated disability. Objective quantification is optimal. It allowed a reliable answer to the main research question: “Does eradicating *H. pylori* have an effect on the primary outcome, hypokinesia of gait?” It also enabled exploration of factors that modify outcome, by interaction or (like ANA status) directly. Greater understanding of the evolution has come from unpicking how different facets respond to the same stimulus. Lumping facets of the syndrome together in a “global” subjective score is valid only if they progress in parallel within-proband, in set proportion between. Biological plausibility of the work lies in a large number of observational studies [2] fitting with the current interventional one.

We show that *Helicobacter* infection can act as a switch in the pathogenic circuitry: marked deterioration in gait accompanied eradication failure, while improvement accompanied success. Taken together, natural and planned experiments incriminate *Helicobacter* specifically. However, a subordinate pathogenic pathway, manifest by increased flexor rigidity, opened up following successful eradication. This reproduction of the natural history of transition from hypokinesia- to rigidity-predominance, together with *Helicobacter*’s ubiquity, suggests it is the natural arbiter for progression.

Small intestinal bacterial overgrowth is a candidate driver for the subordinate route. It is common in IP in the absence of *Helicobacter* [3]. In the case history, flexor rigidity increased on acquiring hydrogen-breath-test positivity, decreased on regaining negativity. However, the increase pre-dated breath-test positivity, and rigidity did not quite return to baseline after negativity was regained. The effect may be load-related, with pathogenic colonization without hydrogen-breath-test positivity. Overgrowth is unlikely to be a bystander in IP: significant numbers of secondary lysosomes are seen in enterocytes, when overgrowth is verified on jejunal aspirate culture [I.T. Bjarnason, R.J. Dobbs, S.M. Dobbs, B. Hudspith, J. O’Donohue, J.D. Sanderson, unpublished observation). Other chronic infections (e.g. urinary and periodontal disease) may be equally harmful to an individual.

### Possible Driving Mechanisms: Autoimmunity and Load-Related’

A specific basal ganglia syndrome, Sydenham’s chorea, has long been associated with spatially remote bacterial (Group A *Streptococcus*) infection. The exceptionality of autoimmune gastritis with *Helicobacter* infection in IP [37] does not exclude cross-reactivity with remote antigens. Autoimmunity may also best explain: (1) benefit of eradicating low-density *Helicobacter* infection (case history); (2) adverse consequence of its persistence in low-density post-treatment (2/4 eradication failures); (3) predictiveness of the immunoblot antibody profile for risk, severity and deterioration of IP, irrespective of anti-urease ELISA-seropositivity [36]. Removal of an autoimmune driver would be expected to result in rapid improvement (case history). Any secondary biomechanical abnormality (e.g. “fixed” knee flexion) may temper the speed of recovery (efficacy study). ANA marked proven failure of eradication. In apparent success, ANA’s association with a brady/hypokinetic response could indicate persistent infection, not detected by the trial methodology. In newly recruited IP probands, in the UK, immunoblot seropositivity (50%, with further 8% equivocal) is currently much more frequent than UBT (30%) or ELISA (35%) positivity [3]. Immunoblot positivity was associated with a relatively higher total lymphocyte count (by, on average 12%). Even an equivocal blot score tended to be (16%). This suggests ongoing infection not just memory. Propensity to low-density infection might be greater where bacterial urease continues to produce ammonia in the face of inhibition of acid secretion by circulating cytokines [44]. Such auto-inhibition could explain low-density gastric infection without atrophy in IP. Ready access to the adaptive immune system might be facilitated by *Helicobacter* gaining access to gastric lymph nodes [45]. Although there is little evidence to support persistence of *H. pylori* in low density in dyspeptic cohorts, the blood profile of IP probands is distinctive, with relative lymphopenia overall, a leftward shift in B-cells and a rightward in natural killer count, compared with controls [3]. Moreover, a low cytotoxic T-cell count appears to flag the risk of eradication failure. Greater incidental anti-microbial usage in IP, if in the context of impaired bacterial clearance, could increase the prevalence of low-density infection.

A likely explanation of non-organism-specific, dose-related, brain damage is activation of microglia to produce inflammatory mediators and neurotoxins, with corresponding suppression of neurotrophic support [9]. With elimination of peripheral infection, activation would be lost, supportive function restored. Systemic inflammation can evade or compromise the blood-brain-barrier to influence the brain’s immune system [2]. Indeed, there are biological gradients between circulating inflammatory markers (cortisol, tumor necrosis factor-α) and objective measures of parkinsonism (gait, sway, psychomotor response) [44,46].

### A Unifying Mitochondrial Hypothesis

Mitochondrial dysfunction is described in IP, both in the substantia nigra and peripherally [2]. In a systematic review [2], we propose that cross-reactivity between *Helicobacter* and mitochondria underlies brady/hypokinesia-predominant IP (Stage 1), and that subsequent acquisition of SIBO stresses mitochondria further, producing a rigidity-predominant picture (Stage 2). On electron microscopy, double-membrane-encapsulated arrays, not associated with rough endoplasmic reticulum, appear to typify duodenal enterocytes of probands with *Helicobacter* infection [47]. They persist, in declining density, for a year or more after successful eradication. Long thin, sometimes complex-branching, mitochondria predominate in probands with SIBO. Hypertrophy, associated with rough endoplasmic reticulum, may compensate for hypofunction. Involvement of proteins made in mitochondria, which regulate mitochondrial function or protect against mitochondrial poisons, are described in different genetic forms of parkinsonism [48–50]. In sporadic IP, an environmental factor, *Helicobacter*, may influence mitochondrial morphology and dynamics. In the overlap disease of sporadic Alzheimer’s, alterations in mitochondrial morphology, distribution, and function appear to influence synaptic function [51].

### Attempt to Accommodate *Helicobacter* Without Paradigm Shift

Speculation about the adverse effect of *Helicobacter* gastritis on totality of levodopa absorption [52] evades a radical move away from the consensus view. The putative mechanism is decreased acid secretion, through inhibition by cytokines or because of atrophic gastritis. However, atrophy more than minimal/mild was unusual in the efficacy study. Perhaps more importantly for absorption, the delayed gastric emptying of IP can improve following *Helicobacter* eradication [37]: this might impact on predictability of response to such a short *t*½ medication.

Levodopa absorption is irrelevant in this study: no proband received it. Moreover, improvement was irrespective of any background anti-parkinsonian medication.

### Summary of Clinical and Research Implications of Work

The size of effect of eradicating *H. pylori* on gait (efficacy study) was clinically relevant (i.e. similar to that of giving levodopa, after overnight deprivation, in probands experiencing end-of-dose effect [18]). It was maintained for at least 2 years, acquisition of increased flexor tone being insufficient to annul improvement. Eradication corrected the marked gait deficit in the case history. There is good reason to rank tremor, where present, as a subsidiary outcome to rigidity: tremor decreased with increase in flexor tone, and *vice versa*. Acquisition of SIBO appeared to dampen the intrusive tremor (case history), regaining hydrogen-breath-test negativity to allow its re-emergence. Balance is critical to realizing functional potential. All three relevant measures (body sway, its dependency on vision, ambulatory breadth-of-base) improved in unison with *Helicobacter* eradication (case history). Individual measures worsened with known or presumed eradication failure (efficacy study): breadth-of-base tended to narrow with known eradication failure, visual dependency increased in the ANA positive. Supine mean arterial pressure fell progressively following *Helicobacter* eradication [53], with a corresponding rise in pulse rate (case history): obesity and fluid retention, associated with elevated serum cortisol [46], may have contributed to initial pressures. Pulse fell with known failure. Cognitive efficiency was unaffected by treating *Helicobacter*.

It is easy to translate work superficially, to the detriment of patients: hence the need to establish robust epidemiological data and guidelines now. Experience so far suggests that a noninvasive test-and-treat policy for *Helicobacter* in IP is inadvisable, due to the dramatic deterioration in parkinsonism which can follow failure. We recommend endoscopic biopsy, to obtain anti-microbial sensitivities at presentation, and in confirming *Helicobacter* eradication, with PCR assay in the culture negative.

Any effect of *Helicobacter* eradication on acquisition of SIBO needs to be defined, as do those of acquiring and eliminating SIBO on rigidity. Common sense life-style changes, involving adequate fluid and fiber intake, and prophylactic bulk-forming/osmotic laxatives need to be well justified to ensure patient uptake.

Old-fashioned meticulous observation in efficacy studies can unravel pathogenetic and therapeutic effects, beyond the scope of intention-to-treat trials. Insights from case studies, where potentially important modifying factors have been recorded and associated with clinical observations (e.g. SIBO with rigidity), further our understanding of pathogenesis. Such detail is difficult to encompass in a single trial. Although randomization will, on average, ensure balance between groups in unrecorded modifying effects, the “noise” they introduce will add to the general variation in outcome, making intervention appear less effective. Clearly, the effects here need to be reproduced, taking into account geographical differences in host and microbial modifying factors. Regulation of systemic inflammation and immunity by host genes might prove equally important to bacterial virulence factors [2], in determining age of presentation with IP.

Our grateful thanks go to Dr Robert J. Owen, Laboratory of Gastrointestinal Pathogens, Health Protection Agency, London, for his help and advice as Head of *Helicobacter* Reference Unit, Drs John Cazabon & Faisal Wahid, Immunology Department, King’s College Hospital, for the cellular immunology, Dr Ron Hutton, What’s Driving Parkinson’s, Psychiatry Research Trust, for his work on the bibliography and Pharmaceutical Sciences, King’s College London, for ongoing academic support.

The Steering Committee comprised of Sir James Black, M.B., F.R.S., Nobel Laureate, James Black Foundation, King’s College; R. Hermon Dowling, F.R.C.P., M.D., President Core, The Charity for Research & Information on Digestive Disorders, 3 St Andrews Place; Ian Talbot, F.R.C.Path., M.D., Histopathology, Northwick Park & St. Mark’s Hospitals, Imperial College; and Jim Wade, F.R.C.Path., M.D., Laboratory Director, Health Protection Agency & Head, Department of Medical Microbiology, King’s College Hospital, London, UK.

The work was funded through the Psychiatry Research Trust, London, which received grants from and the Cecil Pilkington Charitable Trust, the Cyril Corden Trust, the Hayward Foundation, Lord Belstead Charitable Trust, Lord & Lady Lurgan Trust, and the Medlock Charitable Trust, and donations from Brian Newman & Louise Barton, and Michael Howard of Frontier Software PLC, Surrey. Barclays Corporate Social Responsibility Ambassador, Nicholas Smith, co-ordinated a fundraising program with the help of patients and carers. Donations and supply of trial medication were received from Abbott Laboratories, and AstraZeneca.
